# Timing of dexamethasone initiation during controlled ovarian stimulation and live birth after IVF/ICSI: a retrospective cohort study

**DOI:** 10.3389/fendo.2026.1764227

**Published:** 2026-05-13

**Authors:** Ling Zhang, Bin Tang, Yaqian Qin, Xuan Lu, Jia Tang, Ailing Peng

**Affiliations:** 1 Centre for Reproductive Medicine, Changde Hospital, Xiangya School of Medicine, Central South University (The First People's Hospital of Changde City), Changde, China; 2 Department of Gynecology, Changde Hospital, Xiangya School of Medicine, Central South University (The First People’s Hospital of Changde City), Changde, China

**Keywords:** controlled ovarian stimulation, dexamethasone, embryo yield, *in vitro* fertilization, live birth, propensity score

## Abstract

**Background:**

Dexamethasone (DXM) is used as an empirical adjuvant in IVF/ICSI, but evidence for its benefit remains inconsistent, and the clinical importance of initiation timing during controlled ovarian stimulation (COS) is unclear.

**Methods:**

We retrospectively analyzed 644 COS cycles at a tertiary reproductive center. Cycles were classified as no DXM (n = 185), early-follicular DXM (stimulation days 0-4; n = 296), mid-follicular DXM (days 5-7; n = 69), or late/trigger-day DXM (day ≥8 or hCG day; n = 94). DXM was prescribed at the treating physician’s discretion, mainly for cycles considered at increased risk of premature progesterone elevation, and was administered as oral dexamethasone acetate once daily at 0.75-1.125 mg/day, predominantly 0.75 mg/day. Ovarian response, embryo development, and total usable embryos were analyzed in the full COS cohort. Pregnancy outcomes were analyzed among post-transfer cycles, and neonatal outcomes among deliveries. Multivariable logistic regression, propensity score-based inverse probability of treatment weighting, and mediation analysis were used to assess associations and address confounding.

**Results:**

Mid- and late/trigger DXM cycles generated more transferable and frozen embryos and more total usable embryos than cycles without DXM. In the post-transfer cohort, early- and mid-follicular DXM were associated with higher live-birth rates than no DXM, whereas late/trigger DXM showed no clear live-birth advantage. Adjusted and propensity score-weighted models produced consistent estimates. Mediation analysis did not show significant evidence that total usable embryos mediated the association between early DXM and live birth. Neonatal outcomes, including gestational age, birthweight, preterm birth, low birthweight, and congenital malformations, did not differ significantly by DXM timing.

**Conclusions:**

In this retrospective cohort, DXM timing was associated with distinct reproductive outcomes. Early- and mid-follicular DXM were associated with higher live birth after transfer, whereas late/trigger DXM did not show clear clinical benefit. These findings should be interpreted cautiously given the clinically selected nature of DXM use.

## Introduction

Controlled ovarian stimulation (COS) is a cornerstone of *in vitro* fertilization (IVF) and intracytoplasmic sperm injection (ICSI), yet there remains substantial heterogeneity in ovarian response, embryo yield and live birth even among women with similar baseline characteristics. Beyond gonadotropin dose and protocol choice, accumulating evidence suggests that the peri-follicular and peri-implantation inflammatory and endocrine milieu influences follicular development, oocyte competence and endometrial receptivity (1–4). Aberrant immune activation, subtle endocrine dysregulation and low-grade inflammation have all been implicated in suboptimal response to COS, impaired embryo development and recurrent implantation failure in selected patients. These observations have prompted the empirical use of adjuvant therapies aimed at modulating the immune and inflammatory environment (5–9).

Glucocorticoids, including dexamethasone (DXM), are among the most commonly used adjuvants in assisted reproduction. In women with autoimmune disease, thyroid autoimmunity or suspected immune-mediated implantation failure, short courses of glucocorticoids have been proposed to dampen excessive immune activation, improve endometrial receptivity and enhance implantation (10–18). In polycystic ovary syndrome (PCOS) and hyperandrogenic phenotypes, DXM has also been used to suppress adrenal androgen production and potentially improve follicular synchronization (19–23). However, studies evaluating glucocorticoid use in IVF have yielded conflicting results: many are small, heterogeneous in patient selection, timing, dose and duration of treatment, and often underpowered for live birth (10, 11, 13–17). Consequently, major guidelines do not recommend routine glucocorticoid use in unselected IVF populations, and DXM remains a discretionary adjuvant reserved for selected patients at the clinician’s discretion (9, 24–26).

An important but largely underexplored dimension of DXM use during COS is the timing of its initiation. From a biological perspective, early follicular exposure to glucocorticoids might influence follicle recruitment, gonadotropin sensitivity and intra-ovarian cytokine signaling, whereas mid- or late-follicular exposure may primarily modulate peri-ovulatory endocrine dynamics or the peri-implantation immune environment. Clinically, DXM is initiated at variable times across centers and even within the same unit, often driven more by protocol habit or physician preference than by evidence. A few observational studies and small randomized trials have reported on glucocorticoid use in IVF, but most have treated DXM as a binary exposure (yes/no), focused on intermediate endpoints such as implantation or clinical pregnancy, and rarely disentangled the potential impact of DXM timing on ovarian response, embryo yield, live birth and offspring safety (10, 11, 13–17). Concerns also remain about potential adverse effects of glucocorticoids on oocyte or embryo quality and on obstetric and neonatal outcomes, particularly when exposure occurs near conception or in early pregnancy (27–30).

In this retrospective cohort study of women undergoing COS for IVF/ICSI at a tertiary reproductive center, we therefore examined the association between the timing of dexamethasone initiation—categorized as no DXM, early-follicular, mid-follicular and late/trigger-day DXM—and a spectrum of outcomes spanning stimulation parameters, oocyte and embryo yield, total usable embryos per cycle, pregnancy and live birth, and neonatal safety. We used multivariable regression, propensity score–weighted analyses and causal mediation methods to address confounding by indication and to explore whether any observed effects of early DXM on live birth might be mediated through an increased pool of usable embryos. Our primary objective was to determine whether specific DXM timing strategies are associated with differences in post-transfer clinical pregnancy and live birth, while characterizing ovarian response and embryo yield in the full COS cohort and neonatal outcomes in the delivery cohort, and to provide a framework for more rational, evidence-informed use of DXM in modern IVF practice.

## Materials and methods

### Study design and setting

This retrospective cohort study was conducted at the Reproductive Medicine Center of the First People’s Hospital of Changde City. All data were obtained from the electronic medical record and laboratory information systems of the IVF unit. Consecutive controlled ovarian stimulation (COS) cycles that proceeded to oocyte retrieval between January 2022 and June 2025 were screened. The protocol was approved by the Medical Ethics Committee of the First People’s Hospital of Changde City (approval number 2025-439-01) and complied with the Declaration of Helsinki.

### Patient selection

The study population comprised women undergoing COS for IVF or intracytoplasmic sperm injection (ICSI) with either fresh embryo transfer or freeze-all strategies. Inclusion criteria were: reproductive age (approximately 20–45 years), stimulation with a conventional gonadotropin-based COS protocol, availability of baseline endocrine and ovarian reserve parameters (serum FSH, LH, estradiol, progesterone, prolactin, total testosterone, anti-Müllerian hormone [AMH] and antral follicle count [AFC]), and availability of key stimulation and laboratory records. Pregnancy outcomes were analyzed when applicable in cycles that proceeded to embryo transfer, and neonatal variables were analyzed in delivery cycles with available follow-up.

Cycles were excluded if they were cancelled before gonadotropin (Gn) initiation, if core stimulation data (e.g. starting or total gonadotropin dose, trigger information, hCG-day hormones) were missing, or if final oocyte maturation was triggered with regimens for which endocrine measurements were not comparable (e.g. GnRH agonist-only trigger without hCG). Cycles in which experimental adjuvants other than dexamethasone (DXM) were used, or cycles involving preimplantation genetic testing with incomplete outcome data, were also excluded. Each COS cycle was treated as a separate observation in the primary analysis.

### Ovarian stimulation and laboratory procedures

COS was performed using standard GnRH agonist long/short or GnRH antagonist protocols at the discretion of the treating clinician. Recombinant or urinary gonadotropins (Gn) were started at individualized doses based on age, body mass index (BMI), AMH, AFC and any previous response, and adjusted according to serial transvaginal ultrasound and serum estradiol (E2) measurements. Follicular development was monitored from stimulation day 5–6 onwards every 1–3 days. Final oocyte maturation was triggered with hCG or a dual trigger when the leading follicular cohort met predefined criteria (typically ≥3 follicles ≥17–18 mm with an appropriate E2 level) (2).

Transvaginal ultrasound-guided oocyte retrieval was performed 34–36 hours after trigger. Oocytes were inseminated by conventional IVF or ICSI according to semen parameters. Normal fertilization was defined as two pronuclei (2PN) at 16–18 hours. Embryos were assessed on day 2–3 for cleavage stage and morphology, and embryo transfer was performed at cleavage or blastocyst stage according to clinical strategy. Surplus good-quality embryos were cryopreserved at cleavage or blastocyst stage following routine laboratory protocols.

### Dexamethasone administration and exposure groups

DXM was not administered routinely to all COS cycles. In clinical practice at our center, the decision to prescribe DXM and the timing of initiation were individualized by the treating physician according to the patient’s current-cycle follicular development, ovarian responsiveness to gonadotropins, and serial serum progesterone dynamics. DXM was mainly considered for cycles judged to be at increased risk of premature progesterone elevation before trigger, a situation that may cause embryo-endometrium asynchrony and reduce implantation potential. The intended rationale was to modulate endocrine and inflammatory conditions during COS, including potential suppression of aberrant LH secretion through glucocorticoid-mediated hypothalamic-pituitary feedback and attenuation of local ovarian inflammatory activation. Because this was a physician-directed, indication-based intervention rather than a randomized or protocol-mandated treatment, all between-group comparisons were interpreted as observational associations.

The dosing regimen was oral dexamethasone acetate once daily, with a total daily dose of 0.75-1.125 mg; the predominant regimen was 0.75 mg once daily. Treatment was usually continued until the day of hCG trigger or oocyte retrieval, unless contraindications or adverse events required discontinuation (24, 26).

For analysis, each cycle was assigned to one of four mutually exclusive groups according to the timing of DXM initiation relative to the start of gonadotropin administration: No DXM (no DXM at any time); Early DXM (initiation on stimulation days 0–4); Mid DXM (days 5–7); and Late/trigger DXM (day ≥8 of stimulation or on the day of hCG trigger). These windows were chosen to approximate early-, mid- and late-follicular exposure during COS.

### Outcomes related to stimulation, ovarian response and embryo development

Stimulation-related variables included total Gn stimulation days, starting and total Gn dose (IU), and serum LH, E2 and progesterone (P) on the day of hCG trigger. Ovarian hyperstimulation syndrome (OHSS) was defined clinically according to contemporary criteria and coded as present/absent.

Ovarian response and IVF laboratory outcomes included: total punctured follicles and total oocytes at retrieval, oocyte yield (oocytes/punctured follicles, %), number and rate of metaphase II (MII) oocytes, number and rate of 2PN fertilization, and number and rate of cleavage among 2PN embryos. Embryo and blastocyst outcomes included the number of transferable embryos (meeting predefined criteria for transfer or cryopreservation), good-quality cleavage-stage embryos, embryos cultured to blastocyst, blastocysts formed, high-grade blastocysts, and frozen cleavage-stage embryos and blastocysts. For each cycle, total usable embryos were defined as the sum of all transferable and all frozen embryos (cleavage- or blastocyst-stage) generated from that stimulation.

### Clinical, live birth and neonatal outcomes

Biochemical pregnancy was defined as a positive serum β-hCG above the laboratory threshold approximately 14 days after embryo transfer. Clinical pregnancy was defined as at least one intrauterine gestational sac with or without fetal cardiac activity on transvaginal ultrasound. Live birth was defined as delivery of at least one live infant at or beyond 24 weeks’ gestation. Miscarriage was defined as pregnancy loss after biochemical or clinical pregnancy and before 24 weeks’ gestation; where possible, miscarriages were further subclassified as early or late according to gestational age.

The full COS cohort was used for analyses of baseline characteristics, stimulation parameters, ovarian response, embryo development, and total usable embryos. Pregnancy-related outcomes were analyzed in the post-transfer cohort, defined as cycles that proceeded to embryo transfer and had recorded post-transfer follow-up. Within the post-transfer cohort, transfer cycles with negative biochemical pregnancy were coded as clinical pregnancy = no rather than treated as missing. The primary clinical endpoint was live birth in the post-transfer cohort. Key secondary endpoints included biochemical pregnancy and clinical pregnancy in the post-transfer cohort, total usable embryos in the full COS cohort, and detailed embryo/blastocyst quality metrics. For cycles culminating in delivery, neonatal outcomes were assessed in the delivery cohort and included gestational age at delivery, birthweight, preterm delivery (<37 vs ≥37 weeks), low birthweight (<2500 vs ≥2500 g), and congenital malformations. A composite neonatal safety endpoint, ‘any neonatal adverse outcome’, was defined as at least one of: preterm birth, low birthweight, or congenital malformation.

### Statistical analysis

All analyses were performed at the cycle level, but the analytic cohort depended on outcome applicability. The full COS cohort (n=644) was used for baseline, stimulation, ovarian-response, embryo, and total usable embryo analyses. The post-transfer cohort (n=460) was used for biochemical pregnancy, clinical pregnancy, and live-birth analyses. The delivery cohort (n=219) was used for neonatal analyses. Apparent missingness in pregnancy and neonatal variables was largely structural, reflecting non-transfer cycles, freeze-all cycles, cycles with no transferable embryo or no blastocyst, or outcomes only applicable after delivery, rather than missing baseline covariates. Continuous variables were summarized as mean (standard deviation) or median (interquartile range), and categorical variables as counts and percentages. Group differences across DXM timing categories were evaluated using one-way ANOVA for approximately normally distributed continuous variables or Kruskal–Wallis tests for skewed variables; Pearson’s χ² tests or Fisher’s exact tests were used for categorical variables. *Post-hoc* pairwise comparisons were considered exploratory.

To assess associations between DXM timing and pregnancy outcomes, we fitted multivariable logistic regression models with clinical pregnancy or live birth in the post-transfer cohort as binary outcomes. As sensitivity analyses, we additionally refitted the clinical-pregnancy and live-birth models after adjustment for harmonized infertility diagnosis covariates representing infertility type and infertility etiology categories (**Supplementary Table 7**) and, separately, after adjustment for within-cycle progesterone change (ΔP = hCG-day progesterone minus basal progesterone; **Supplementary Figure 7**; **Supplementary Table 8**).

Because DXM use and timing were not randomized, we additionally used propensity score (PS)–based methods to address confounding by indication. For any DXM vs no DXM, PS were estimated via logistic regression including age, BMI, AMH, AFC and basal FSH, LH, E2, P, prolactin and total testosterone. Inverse probability of treatment weights (IPTW) were calculated as 1/PS for treated cycles and 1/(1−PS) for untreated cycles; extreme weights were truncated at the 99th percentile. Covariate balance before and after weighting was assessed using standardized mean differences (SMD), with |SMD|<0.1 indicating acceptable balance. IPTW-weighted logistic models for live birth were then fitted and compared with conventional multivariable models. In separate pairwise analyses (early vs no DXM, mid vs no DXM, late/trigger vs no DXM), we constructed PS and IPTW with the same covariates and obtained timing-specific IPTW-adjusted ORs for live birth (31).

Subgroup analyses examined early DXM vs no DXM within strata defined by age (<30 vs ≥30 years) and by AMH (below vs above the cohort median), using multivariable logistic models with the same covariates. Formal interaction terms (e.g. DXM group × age group, DXM group × AMH group) were evaluated in additional models; these analyses were prespecified as exploratory and no multiplicity correction was applied to interaction P values.

To explore whether the effect of early DXM on live birth was partly mediated through embryo yield, we performed causal mediation analysis using the mediate function in the R mediation package (32–34). Among cycles with early DXM or no DXM, total usable embryos were modelled as a function of early DXM and clinical covariates, and live birth as a function of early DXM, total usable embryos and the same covariates. We estimated the average causal mediation effect (ACME), average direct effect (ADE), total effect and proportion mediated, with 95% CIs obtained via nonparametric bootstrapping (1,000 simulations). Results were reported on the log-odds scale.

Given the number of secondary endpoints, particularly among stimulation and embryo measures, we considered these analyses exploratory and did not apply strict family-wise error correction. As a sensitivity analysis, Benjamini–Hochberg false discovery rate (FDR)–adjusted P values were calculated for Kruskal–Wallis tests on selected key variables, and the main findings for total usable embryos and live birth remained robust at FDR <0.1. All statistical analyses were performed using **R** (version 4.4.2; R Foundation for Statistical Computing, Vienna, Austria).

## Results

### Study population and overall cycle outcomes

**Figure 1:**
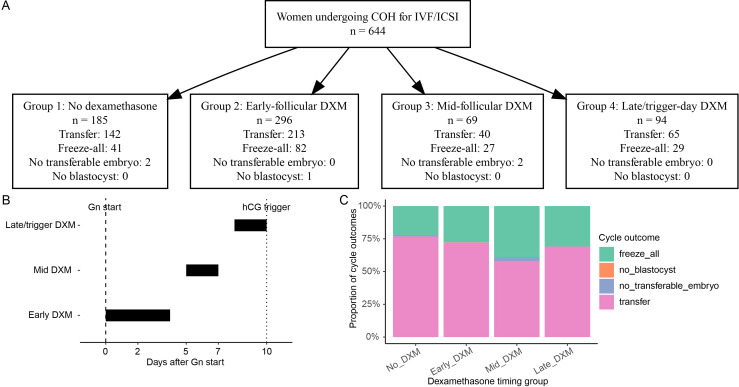
Study flow and classification of dexamethasone timing. **(A)** Flow diagram of women undergoing COS, controlled ovarian stimulation for IVF/ICSI and allocation to four exposure groups: no DXM, dexamethasone, early-follicular DXM, mid-follicular DXM and late/trigger-day DXM. Numbers of cycles proceeding to transfer, freeze-all, no transferable embryo and no blastocyst are shown for each group. **(B)** Schematic representation of the timing windows for DXM initiation relative to gonadotropin (Gn) start and hCG trigger, with early, mid and late/trigger DXM defined as initiation on stimulation days 0–4, 5–7 and ≥8 or on hCG day, respectively. **(C)** Distribution of cycle outcomes (transfer, freeze-all, no transferable embryo, no blastocyst) by DXM timing group, expressed as proportions of cycles. .

A total of 644 stimulation cycles were included in the full COS cohort: 185 without dexamethasone (No DXM), 296 with early-follicular DXM (Early DXM), 69 with mid-follicular DXM (Mid DXM), and 94 with late/trigger-day DXM (Late/trigger DXM) (**Figure 1A**). This full cohort was used for baseline, stimulation, ovarian-response, embryo-development, and total usable embryo analyses. Among these cycles, 460 proceeded to embryo transfer and had recorded post-transfer outcome data, forming the post-transfer cohort for biochemical pregnancy, clinical pregnancy, and live-birth analyses. A total of 219 delivery cycles comprised the delivery cohort for neonatal analyses (**Supplementary Table 6**). Variable availability and derivation of the analytic cohorts are summarized in **Supplementary Table 6**. Across groups, most cycles proceeded to oocyte retrieval and embryo transfer, whereas cycle cancellation due to poor response or lack of transferable embryos was infrequent and comparable between groups (**Figure 1C**). Apparent missingness in pregnancy and neonatal variables was largely structural and reflected cohort applicability rather than random baseline covariate missingness.

The schematic in **Figure 1B** summarizes the intended timing of DXM initiation relative to gonadotropin (Gn) start and hCG trigger, with ‘early’ defined as stimulation days 0–4, ‘mid’ as days 5–7, and ‘late/trigger’ as day ≥8 or the hCG day. Consistent with this day-based exposure definition, dominant follicle diameter at DXM initiation differed markedly across the early, mid, and late/trigger DXM groups and showed high concordance with the expected follicular developmental stage (**Supplementary Table 9**).

### Baseline characteristics

Baseline demographic, infertility, and ovarian reserve characteristics were broadly similar across the four groups (**Table 1**). Mean age, duration of infertility, AMH level, and antral follicle count did not differ significantly between groups, whereas BMI differed modestly across groups (**Table 1**). Basal FSH, E2, P, PRL and total testosterone were also comparable. A modest but statistically significant difference was observed for basal LH (P≈0.001), with slightly lower LH in the mid-follicular group and somewhat higher values in the late/trigger group, but the absolute differences were small.

**Table 1:** Baseline characteristics of patients according to timing of dexamethasone administration.

Variable	No DXM (n=185)	Early DXM (n=296)	Mid DXM (n=69)	Late/trigger DXM (n=94)	P value
Number of cycles	185	296	69	94	–
Age (years)	30.38 (3.04)	29.92 (2.95)	30.36 (3.07)	30.34 (3.19)	0.317
BMI (kg/m²)	24.09 (3.69)	23.28 (3.41)	22.43 (3.10)	22.25 (2.98)	<0.001
Duration of infertility (years)	3.15 (2.03)	3.10 (2.07)	3.03 (2.20)	3.48 (2.87)	0.494
AMH (ng/mL)	5.14 (2.88)	5.25 (2.63)	5.40 (2.49)	5.28 (2.77)	0.915
Antral follicle count	19.01 (4.82)	19.37 (4.83)	18.77 (5.15)	18.97 (4.90)	0.727
Basal FSH (IU/L)	6.76 (1.64)	6.84 (1.56)	6.33 (1.61)	6.90 (1.59)	0.092
Basal LH (IU/L)	5.15 (3.03)	5.02 (2.54)	4.22 (1.94)	5.95 (3.55)	0.001
Basal E2 (ng/mL)	33.93 (12.51)	34.70 (12.29)	32.40 (13.07)	36.54 (10.89)	0.166
Basal P (ng/mL)	0.64 (1.07)	0.64 (0.41)	0.60 (0.41)	0.68 (0.59)	0.903
Basal PRL (ng/mL)	15.00 (7.85)	15.30 (7.57)	15.97 (6.11)	16.25 (9.95)	0.576
Basal T (ng/mL)	1.85 (5.55)	2.85 (15.49)	1.59 (0.65)	1.51 (0.62)	0.608
Primary infertility	96 (51.9)	162 (54.7)	36 (52.2)	47 (50.0)	0.914
Primary infertility post-ART ectopic	0 (0.0)	1 (0.3)	0 (0.0)	0 (0.0)	–
Secondary infertility	89 (48.1)	133 (44.9)	33 (47.8)	47 (50.0)	–
Male factor	0 (0.0)	3 (1.0)	0 (0.0)	0 (0.0)	0.601
Other diagnosis	14 (7.6)	18 (6.1)	8 (11.6)	4 (4.3)	–
PCOS	13 (7.0)	32 (10.8)	5 (7.2)	8 (8.5)	–
PID sequelae	62 (33.5)	82 (27.7)	18 (26.1)	33 (35.1)	–
Tubal factor	96 (51.9)	160 (54.1)	38 (55.1)	49 (52.1)	–
Uterine factor	0 (0.0)	1 (0.3)	0 (0.0)	0 (0.0)	–

Data are presented as mean (standard deviation) or n (%). P values are from one-way ANOVA for continuous variables and χ² tests for categorical variables.

Baseline demographic, infertility and ovarian reserve characteristics of women undergoing COS, controlled ovarian stimulation for IVF/ICSI, stratified by DXM, dexamethasone timing group (no DXM, early DXM, mid DXM, late/trigger DXM). Values are presented as mean (standard deviation) for continuous variables and n (%) for categorical variables. P values are derived from one-way ANOVA for continuous variables and χ² tests for categorical variables.

The distribution of infertility type (primary vs secondary) and etiology (male factor, tubal factor, PCOS, PID sequelae, uterine factor, other) was balanced, with no clear enrichment of any specific infertility diagnosis in a given DXM timing group (**Table 1**).

### Ovarian stimulation and hCG-day endocrine characteristics

**Figure 2:**
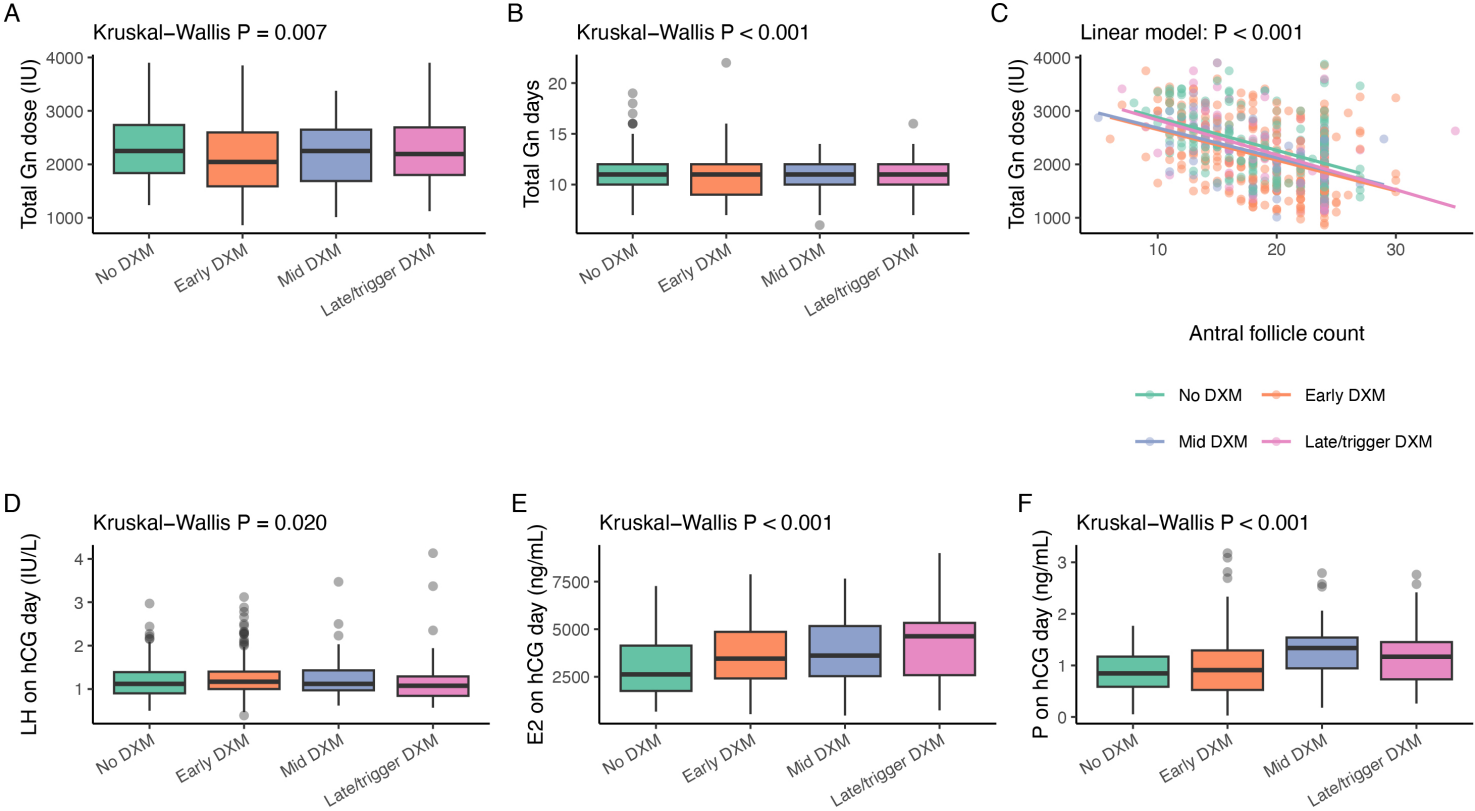
Ovarian stimulation characteristics and hCG-day endocrine profile in the full COS cohort according to dexamethasone timing. **(A)** Total Gn dose (IU) per cycle by DXM timing group. **(B)** Total Gn stimulation days. **(C)** Relationship between AFC, antral follicle count and total Gn dose, with separate regression lines for each DXM timing group. **(D–F)** Serum LH, E2estradiol, and P, progesterone concentrations on the day of hCG trigger. Boxplots show medians and interquartile ranges; P values are from Kruskal–Wallis tests for overall group differences, and from a linear model in panel **(C)**.

Key stimulation parameters in the full COS cohort are summarized in **Table 2** and **Figures 2A–F**. Total Gn stimulation days differed significantly across DXM timing groups and were generally shorter in the DXM groups than in the no-DXM group (Kruskal–Wallis P < 0.001; **Figure 2B**; **Table 2**). Gn starting dose also differed modestly between groups (P = 0.046), whereas total Gn dose was lower overall in the DXM groups than in the no-DXM group (P = 0.007; **Figure 2A**; **Table 2**).

**Table 2:** Ovarian stimulation and hCG-day endocrine characteristics in the full COS cohort according to dexamethasone timing.

Variable	No DXM (n=185)	Early DXM (n=296)	Mid DXM (n=69)	Late/trigger DXM (n=94)	P value
n	185	296	69	94	
Gn total days (mean (SD))	11.32 (1.90)	10.65 (1.83)	10.81 (1.59)	10.81 (1.54)	<0.001
Gn start dose (mean (SD))	185.27 (70.08)	176.98 (67.76)	200.00 (67.99)	185.80 (72.09)	0.046
Gn total dose (mean (SD))	2323.49 (621.25)	2114.27 (647.79)	2190.88 (559.76)	2244.35 (631.54)	0.007
hCG-day LH (mean (SD))	1.19 (0.40)	1.25 (0.39)	1.24 (0.45)	1.17 (0.52)	0.02
hCG-day E2 (mean (SD))	2976.41 (1540.01)	3598.03 (1463.94)	3871.70 (1723.99)	4134.90 (1741.85)	<0.001
hCG-day P (mean (SD))	0.87 (0.36)	0.94 (0.53)	1.27 (0.50)	1.14 (0.50)	<0.001
OHSS (%): No	180 (97.3)	289 (97.6)	69 (100.0)	92 (97.9)	0.687
OHSS (%): Yes	5 (2.7)	7 (2.4)	0 (0.0)	2 (2.1)	–

Data are presented as mean (standard deviation). P values are from one-way ANOVA or Kruskal–Wallis tests, as appropriate.

Stimulation parameters and endocrine profile on the day of hCG trigger across DXM, dexamethasone timing groups in the full COS cohort, including total stimulation days, starting and total Gn, gonadotropin doses, hCG-day serum LH; E2, estradiol; P, progesterone, and OHSS, ovarian hyperstimulation syndrome. Values are presented as mean (standard deviation) or n (%). P values are derived from one-way ANOVA or Kruskal–Wallis tests for continuous variables and χ² or Fisher’s exact tests for categorical variables, as appropriate.

On the hCG day, LH concentrations showed a small but statistically significant difference across groups (P = 0.020), while E2 and progesterone concentrations were progressively higher in the DXM groups, particularly in the mid and late/trigger groups (both P < 0.001; **Figures 2D–F**; **Table 2**). As expected, total Gn dose decreased with increasing AFC, and this overall relationship remained evident across all DXM timing groups (**Figure 2C**). OHSS was rare in this cohort and did not differ significantly between groups (Fisher’s exact P = 0.687; **Table 2**).

### Ovarian response and fertilization outcomes

**Figure 3:**
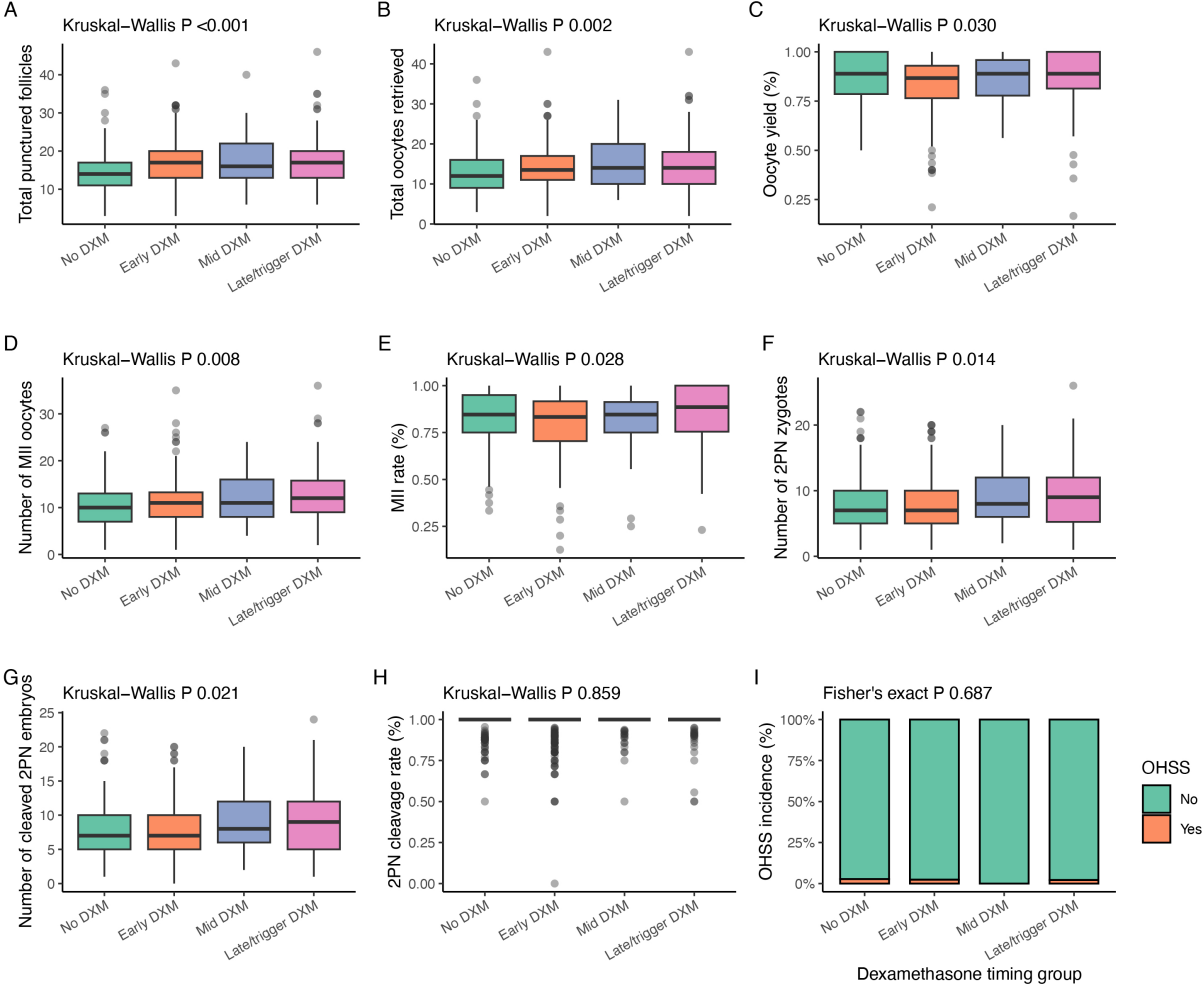
Ovarian response, fertilization outcomes, and OHSS incidence in the full COS cohort by dexamethasone timing. **(A)** Total number of punctured follicles on the day of oocyte retrieval. **(B)** Total number of oocytes retrieved. **(C)** Oocyte yield (oocytes retrieved/punctured follicles, %). **(D)** Number of MII, metaphase II oocytes. **(E)** MII rate (MII oocytes/total oocytes, %). **(F)** Number of 2PN zygotes. **(G)** Number of cleaved 2PN embryos. **(H)** 2PN cleavage rate (cleaved 2PN embryos/2PN zygotes, %). Panels **(A–H)** are presented as boxplots with Kruskal–Wallis P values. **(I)** Proportion of cycles with OHSS, ovarian hyperstimulation syndrome in each group, displayed as stacked bars (yes/no) with Fisher’s exact P value.

Ovarian response at oocyte retrieval is shown in **Table 3** and **Figures 3A–C**. Total punctured follicles and total oocytes retrieved differed significantly across groups, with higher counts generally observed in the DXM groups than in the no-DXM group (both P ≤ 0.002; **Figures 3A, B**; **Table 3**). Oocyte yield remained high in all groups but showed a modest overall difference (P = 0.030; **Figure 3C**; **Table 3**). The number of MII oocytes and the MII rate also differed across groups (P = 0.008 and P = 0.028, respectively; **Figures 3D, E**; **Table 3**), with the lowest MII rate observed in the early-DXM group.

**Table 3:** Ovarian response and fertilization outcomes in the full COS cohort according to dexamethasone timing.

Variable	No DXM (n=185)	Early DXM (n=296)	Mid DXM (n=69)	Late/trigger DXM (n=94)	P value
n	185	296	69	94	
D0 punctured follicles (mean (SD))	14.52 (5.44)	16.87 (5.63)	17.55 (6.65)	17.43 (6.81)	<0.001
D0 oocytes total (mean (SD))	12.66 (5.33)	14.08 (5.39)	15.20 (6.30)	15.23 (7.03)	0.002
Oocyte yield (mean (SD))	0.87 (0.13)	0.83 (0.14)	0.86 (0.12)	0.86 (0.15)	0.03
D0 MII oocytes number (mean (SD))	10.52 (4.67)	11.17 (4.81)	12.30 (5.28)	12.67 (6.07)	0.008
MII rate (mean (SD))	0.84 (0.15)	0.80 (0.16)	0.82 (0.15)	0.84 (0.15)	0.028
2PN number (mean (SD))	7.83 (4.11)	8.08 (3.81)	9.04 (4.18)	9.38 (4.79)	0.014
2PN rate (mean (SD))	0.74 (0.19)	0.74 (0.17)	0.75 (0.19)	0.74 (0.17)	0.904
Cleaved 2PN number (mean (SD))	7.62 (4.07)	7.79 (3.73)	8.75 (4.15)	9.07 (4.72)	0.021
2PN cleavage rate (mean (SD))	0.97 (0.07)	0.96 (0.10)	0.96 (0.08)	0.96 (0.09)	0.859

Data are presented as mean (standard deviation). P values are from one-way ANOVA or Kruskal–Wallis tests, as appropriate.

Ovarian response at oocyte retrieval and early fertilization outcomes stratified by DXM, dexamethasone timing group, including number of punctured follicles and oocytes retrieved, oocyte yield, number and rate of MII, metaphase II oocytes, number and rate of 2PN fertilization, and number and rate of cleavage among 2PN embryos. Values are presented as mean (standard deviation). P values are derived from one-way ANOVA or Kruskal–Wallis tests, as appropriate.

Fertilization and cleavage metrics are summarized in **Figures 3F–H**. The numbers of 2PN zygotes and cleaved 2PN embryos were higher in the mid- and late-DXM groups than in the no-DXM and early-DXM groups (P = 0.014 and P = 0.021, respectively; **Figures 3F, G**; **Table 3**). In contrast, 2PN fertilization rate and 2PN cleavage rate did not differ significantly across groups (P = 0.904 and P = 0.859; **Table 3**; **Figure 3H**). OHSS incidence remained very low and did not differ between groups (**Figure 3I**), supporting the safety of DXM with respect to hyperstimulation.

### Embryo development and total usable embryos

**Figure 4:**
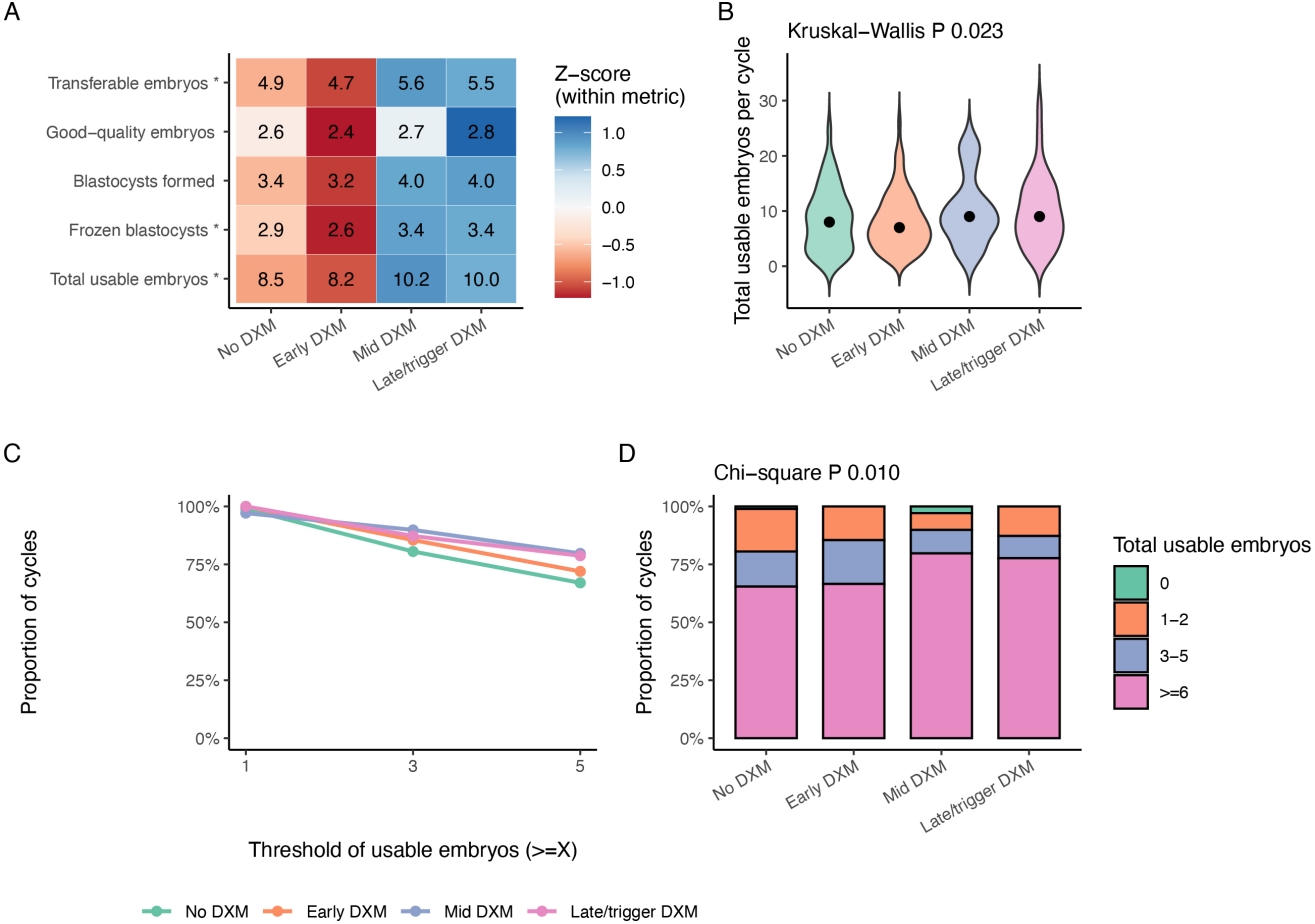
Embryo development and total usable embryos in the full COS cohort according to dexamethasone timing. **(A)** Heatmap of mean values (displayed) and within-metric Z-scores (color scale) for transferable embryos, good-quality cleavage-stage embryos, blastocysts formed, frozen blastocysts and total usable embryos per cycle in each DXM timing group; asterisks indicate metrics with significant Kruskal–Wallis P values. **(B)** Distribution of total usable embryos per cycle (sum of all transferable and frozen cleavage/blastocyst embryos) shown as violin plots with median points; overall P value from Kruskal–Wallis test. **(C)** Probability of achieving at least 1, 3 or 5 usable embryos per cycle by DXM timing group. **(D)** Stacked bar chart showing the proportion of cycles with 0, 1–2, 3–5 or ≥6 total usable embryos; overall χ² P value is shown. .

Embryo and blastocyst outcomes are presented in **Table 4** and **Figures 4A–D**. The heatmap in **Figure 4A** depicts group-level z-scores for five key embryo metrics: transferable embryos, good-quality embryos, blastocysts formed, frozen blastocysts, and total usable embryos. Compared with no DXM, mid and late DXM groups tended to have higher mean numbers of transferable embryos, frozen cleavage embryos, frozen blastocysts, and total usable embryos, whereas the early-DXM group was intermediate (**Table 4**; **Figure 4A**). The total number of usable embryos per cycle differed significantly across groups (Kruskal–Wallis P = 0.023; **Figure 4B**; **Table 4**), with the highest means observed in the mid and late DXM groups (approximately 10.2 and 10.0 per cycle) compared with no DXM (approximately 8.5) and early DXM (approximately 8.2).

**Table 4:** Embryo, blastocyst, and cryopreservation outcomes in the full COS cohort according to dexamethasone timing.

Variable	No DXM (n=185)	Early DXM (n=296)	Mid DXM (n=69)	Late/trigger DXM (n=94)	P value
n	185	296	69	94	
Transferable embryos (mean (SD))	4.88 (2.72)	4.67 (2.44)	5.57 (3.03)	5.52 (3.04)	0.033
Good-quality embryos (mean (SD))	2.62 (2.03)	2.44 (1.87)	2.67 (2.18)	2.84 (2.20)	0.56
Blastocysts cultured (mean (SD))	4.96 (3.37)	5.11 (3.48)	6.00 (3.69)	5.85 (4.38)	0.128
Blastocysts formed (mean (SD))	3.42 (2.78)	3.21 (2.55)	3.96 (3.07)	3.98 (3.17)	0.154
High-grade blastocysts (mean (SD))	1.25 (1.62)	1.10 (1.50)	1.33 (1.69)	1.48 (1.91)	0.448
Frozen cleavage embryos (mean (SD))	0.79 (0.93)	0.96 (0.96)	1.20 (0.93)	1.10 (1.01)	0.008
Frozen blastocysts (mean (SD))	2.87 (2.55)	2.57 (2.38)	3.41 (2.90)	3.38 (2.83)	0.033
Total usable embryos (mean (SD))	8.54 (5.70)	8.20 (5.18)	10.17 (6.32)	10.00 (6.33)	0.023

Data are presented as mean (standard deviation). P values are from one-way ANOVA or Kruskal–Wallis tests, as appropriate.

Embryo and blastocyst metrics and cryopreservation outcomes by DXM, dexamethasone timing group, including numbers of transferable embryos, good-quality cleavage-stage embryos, embryos cultured to blastocyst, blastocysts formed, high-grade blastocysts, frozen cleavage-stage embryos, frozen blastocysts and total usable embryos per cycle (defined as all transferable plus all frozen embryos from the stimulation). Values are presented as mean (standard deviation). P values are derived from one-way ANOVA or Kruskal–Wallis tests, as appropriate.

Transferable embryos, frozen cleavage embryos, and frozen blastocysts also differed significantly across groups, whereas good-quality embryos, blastocysts cultured, blastocysts formed, and high-grade blastocysts did not show statistically significant between-group differences (**Table 4**). When examined as thresholds, the proportions of cycles achieving at least 1, 3, or 5 usable embryos remained higher in the mid- and late-DXM groups than in the no-DXM group (**Figure 4C**). Categorizing cycles by total usable embryos (0, 1–2, 3–5, ≥6) illustrated the same pattern more clearly, with the largest fraction of cycles achieving ≥6 usable embryos in the mid- and late-DXM groups (χ² P = 0.010; **Figure 4D**).

### Pregnancy and live birth outcomes

**Figure 5:**
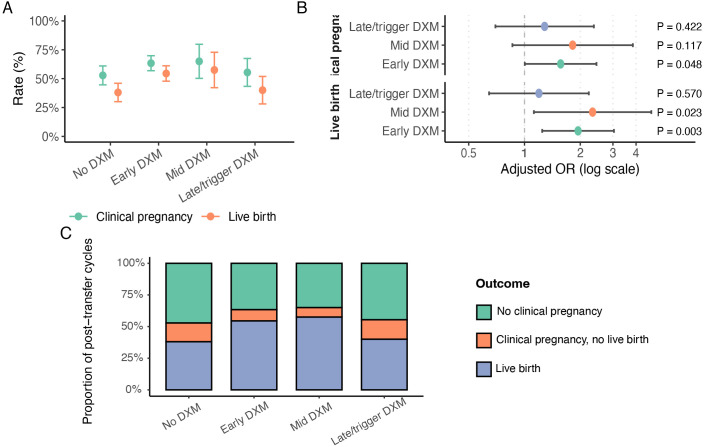
Clinical pregnancy and live birth among post-transfer cycles by dexamethasone timing. **(A)** Clinical pregnancy and live-birth rates among post-transfer cycles in each DXM timing group, presented as percentages with 95% confidence intervals. **(B)** Adjusted ORs, odds ratios with 95% confidence intervals for clinical pregnancy and live birth comparing early, mid, and late/trigger DXM with no DXM (reference), from multivariable logistic models fitted in the post-transfer cohort and adjusted for age, BMI, AMH, AFC, and number of embryos transferred; P values are shown for each contrast. **(C)** Composition of post-transfer cycle outcomes (no clinical pregnancy, clinical pregnancy without live birth, live birth) by DXM timing group, expressed as proportions of post-transfer cycles. .

Post-transfer pregnancy and live-birth outcomes are summarized in **Table 5A** and **Figure 5**. In the post-transfer cohort (No DXM n=142, Early DXM n=213, Mid DXM n=40, and Late/trigger DXM n=65), clinical pregnancy rates were 52.8%, 63.4%, 65.0%, and 55.4%, respectively, whereas live-birth rates were 38.0%, 54.5%, 57.5%, and 40.0%.

**Table 5:** **A.** Post-transfer pregnancy and live-birth outcomes according to dexamethasone timing.

Outcome	No DXM (n=142)	Early DXM (n=213)	Mid DXM (n=40)	Late/trigger DXM (n=65)	P value
Number of post-transfer cycles	142	213	40	65	
Biochemical pregnancy: no	57 (40.1)	68 (31.9)	13 (32.5)	25 (38.5)	0.402
Biochemical pregnancy: yes	85 (59.9)	145 (68.1)	27 (67.5)	40 (61.5)	
Clinical pregnancy: no	67 (47.2)	78 (36.6)	14 (35.0)	29 (44.6)	0.181
Clinical pregnancy: yes	75 (52.8)	135 (63.4)	26 (65.0)	36 (55.4)	
Live birth: 0 live birth	88 (62.0)	97 (45.5)	17 (42.5)	39 (60.0)	0.006
Live birth: >=1 live birth	54 (38.0)	116 (54.5)	23 (57.5)	26 (40.0)	
Miscarriage among clinical pregnancies: no	66 (88.0)	120 (88.9)	24 (92.3)	29 (80.6)	0.512
Miscarriage among clinical pregnancies: yes	9 (12.0)	15 (11.1)	2 (7.7)	7 (19.4)	–
B. Neonatal outcomes among deliveries according to dexamethasone timing.					
Outcome	No DXM (n=54)	Early DXM (n=116)	Mid DXM (n=23)	Late/trigger DXM (n=26)	P value
Number of deliveries	54	116	23	26	
Gestational age at delivery (weeks)	37.31 (1.97)	37.39 (1.89)	37.57 (1.27)	37.96 (1.62)	0.484
Birthweight (g)	3293.71 (433.31)	3122.64 (521.88)	3218.24 (447.82)	3271.82 (514.41)	0.281
Congenital malformations, mean (SD)	0.00 (0.00)	0.00 (0.00)	0.00 (0.00)	0.00 (0.00)	–

Data are presented as n (%) or mean (standard deviation). P values are from χ² or Fisher’s exact tests for categorical variables and one-way ANOVA or Kruskal–Wallis tests for continuous variables, as appropriate. **Table 5A** summarizes post-transfer pregnancy and live-birth outcomes in each DXM, dexamethasone timing group, including biochemical pregnancy, clinical pregnancy, live birth (0 vs ≥1 live birth), and miscarriage. Outcomes are presented among post-transfer cycles. Within the post-transfer cohort, transfer cycles with negative biochemical pregnancy were coded as clinical pregnancy = no rather than treated as missing. Miscarriage was analyzed among clinical pregnancies only. Data are presented as n (%) or mean (standard deviation). P values are from χ² tests or Fisher’s exact tests for categorical variables and one-way ANOVA or Kruskal–Wallis tests for continuous variables, as appropriate.

In multivariable logistic regression adjusting for age, BMI, AMH, AFC, and number of embryos transferred, Early DXM was associated with higher odds of clinical pregnancy (adjusted OR 1.567, 95% CI 1.004 to 2.447, P = 0.048) and live birth (adjusted OR 1.946, 95% CI 1.246 to 3.038, P = 0.003) compared with No DXM. Mid DXM showed a numerically higher odds of clinical pregnancy that did not reach statistical significance (adjusted OR 1.819, 95% CI 0.861 to 3.843, P = 0.117), but was associated with higher odds of live birth (adjusted OR 2.331, 95% CI 1.123 to 4.835, P = 0.023). Late/trigger DXM did not show a clear advantage for either clinical pregnancy (adjusted OR 1.285, 95% CI 0.697 to 2.368, P = 0.422) or live birth (adjusted OR 1.197, 95% CI 0.644 to 2.224, P = 0.570).

Among clinical pregnancies, miscarriage was infrequent and miscarriage rates did not differ significantly across groups. The outcome-composition plot showed higher proportions of both clinical pregnancy and live birth in the Early and Mid DXM groups, with the clearest separation observed for live birth.

Diagnosis-adjusted sensitivity analyses yielded effect estimates that were materially unchanged relative to the prespecified base models (**Supplementary Table 7**). As shown in **Supplementary Figure 7**, within-cycle progesterone change (ΔP) differed significantly across DXM timing groups (Kruskal–Wallis P < 0.001). However, additional adjustment for ΔP did not materially alter the associations with live birth; in the most conservative model additionally adjusting for both ΔP and diagnosis covariates, the association between early DXM and clinical pregnancy was modestly attenuated and became borderline, whereas the live-birth associations for early and mid DXM remained statistically significant (**Supplementary Table 8**). In exploratory linear models, adjustment for ΔP attenuated the mid- and late-DXM associations with total usable embryos, suggesting that late-follicular progesterone dynamics may partly contribute to embryo-yield differences.

### analyse Propensity score–weighted and mediation s

**Figure 6:**
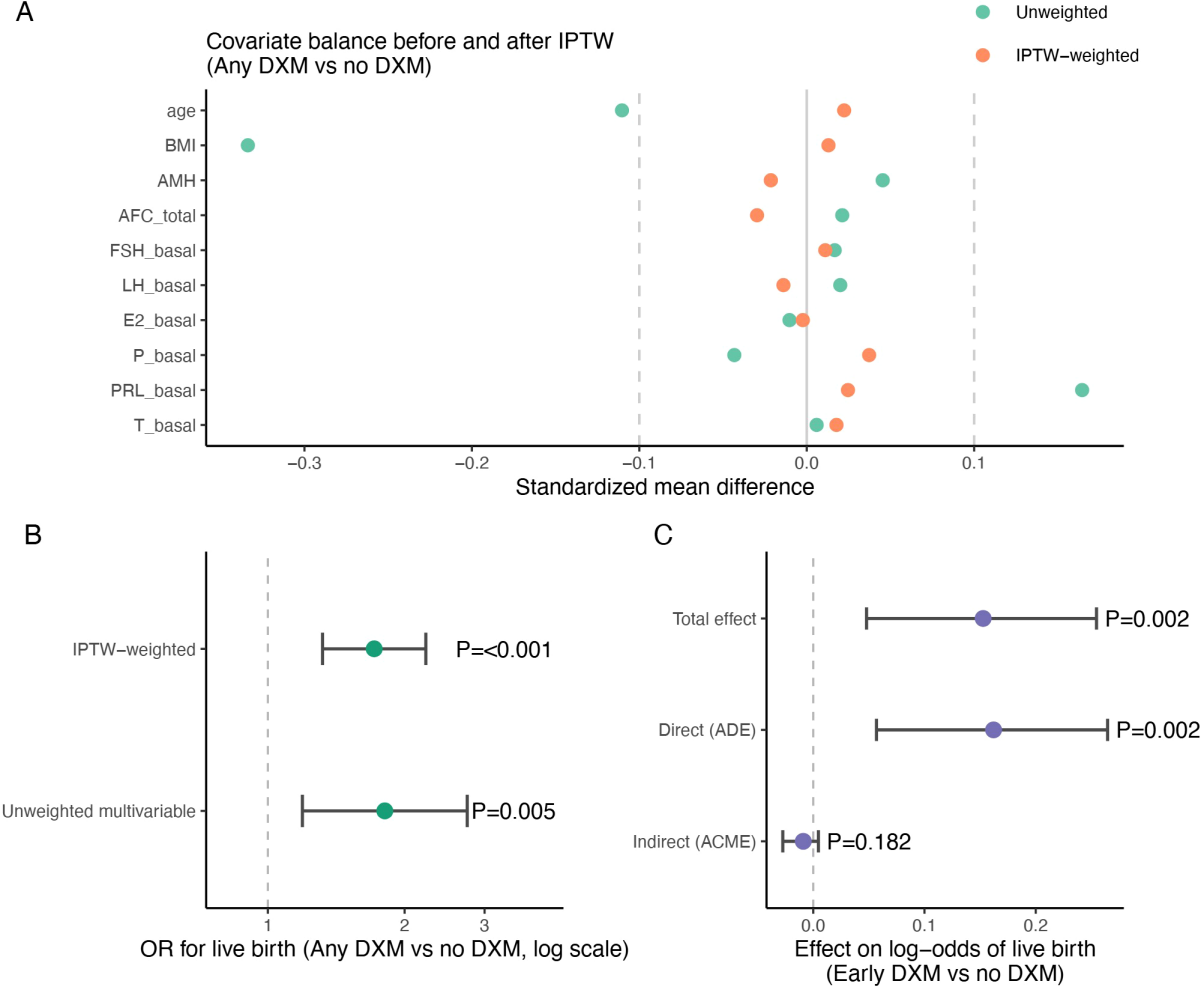
Propensity score balance, IPTW effects and mediation analysis. **(A)** SMD, Standardized mean differences for baseline covariates before (unweighted) and after IPTW in the comparison of any DXM vs no DXM. Each point represents the SMD for a covariate; dashed lines at ±0.1 indicate the threshold for acceptable balance. **(B)** ORs for live birth comparing any DXM vs no DXM from an unweighted multivariable logistic model and an IPTW-weighted model (marginal effect), with 95% CIs on the log scale and corresponding P values. **(C)** Results of causal mediation analysis for early DXM vs no DXM, showing the estimated indirect effect (ACME, average causal mediation effect), direct effect (ADE, average direct effect) and total effect on the log-odds scale with 95% CIs and P values.

To mitigate confounding by indication, we first modelled the probability of receiving dexamethasone using logistic regression including age, BMI, AMH, AFC, and basal FSH, LH, E2, P, PRL and total testosterone. For the comparison of any DXM use versus no DXM, inverse probability of treatment weights (IPTW) were constructed from these propensity scores. After weighting, standardized mean differences for all covariates were reduced to within ±0.1, indicating good covariate balance between DXM and non-DXM cycles (**Figure 6A**; **Supplementary Figure 1**).

**Figure 7:**
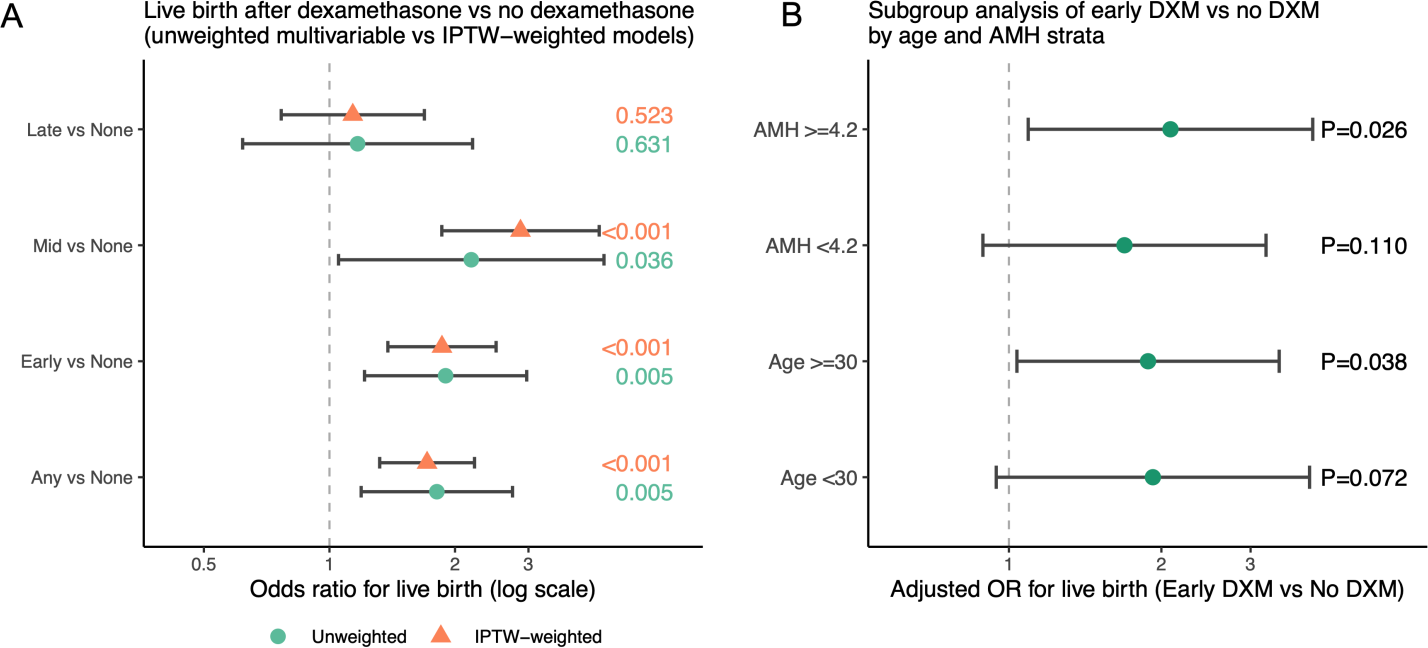
Propensity score–weighted estimates and subgroup effects for live birth. **(A)** Forest plot of ORs for live birth comparing any DXM vs no DXM and, separately, early, mid and late/trigger DXM vs no DXM, derived from unweighted multivariable logistic models (adjusted for age, BMI, AMH, AFC and embryos transferred) and from IPTW, inverse probability of treatment–weighted models based on propensity scores. Points represent ORs and horizontal bars represent 95% CIs on the log scale; P values are shown for each estimate. **(B)** Subgroup forest plot of adjusted ORs for live birth comparing early DXM vs no DXM within strata defined by age (<30 vs ≥30 years) and AMH (<4.2 vs ≥4.2 ng/mL), with 95% CIs and P values for each subgroup. .

In the unweighted multivariable logistic model, any DXM use showed higher odds of live birth compared with no DXM after adjustment for age, BMI, AMH, AFC and number of embryos transferred. A similarly sized or slightly stronger association was observed in the IPTW-weighted model (**Figure 6B**), suggesting that residual confounding alone is unlikely to fully explain the observed benefit. We then extended the propensity score approach to pairwise comparisons of each DXM timing group versus no DXM. In IPTW-weighted logistic models, early- and mid-follicular DXM were consistently associated with increased odds of live birth relative to no DXM, whereas late/trigger-day DXM did not demonstrate a clear advantage (**Figure 7A**; **Supplementary Figure 2**).

To explore potential effect modification, we evaluated early DXM vs no DXM within clinically relevant strata of age (<30 vs ≥30 years) and AMH (below vs above the cohort median). As illustrated in **Figure 7B** and **Supplementary Figure 4**, the point estimates suggested that the favorable association between early DXM and live birth may be more pronounced in younger women and in those with higher AMH; however, confidence intervals were wide and formal interaction tests did not reach statistical significance, and these subgroup findings should therefore be interpreted cautiously.

Finally, we performed a mediation analysis to interrogate whether the apparent benefit of early DXM on live birth is partially mediated through an increased pool of usable embryos. Restricting to cycles with early DXM or no DXM, we modelled total usable embryos as a mediator and live birth as the outcome, adjusting for the same clinical covariates. The mediation analysis did not show statistically significant evidence for an indirect effect through total usable embryos (ACME), whereas the direct effect (ADE) of early DXM on live birth remained statistically significant (**Figure 6C**; **Supplementary Table 3**). These findings suggest that any mediation through total usable embryos was limited and that the observed association between early DXM and live birth was not strongly explained by embryo number alone.

### Neonatal outcomes and safety

**Figure 8:**
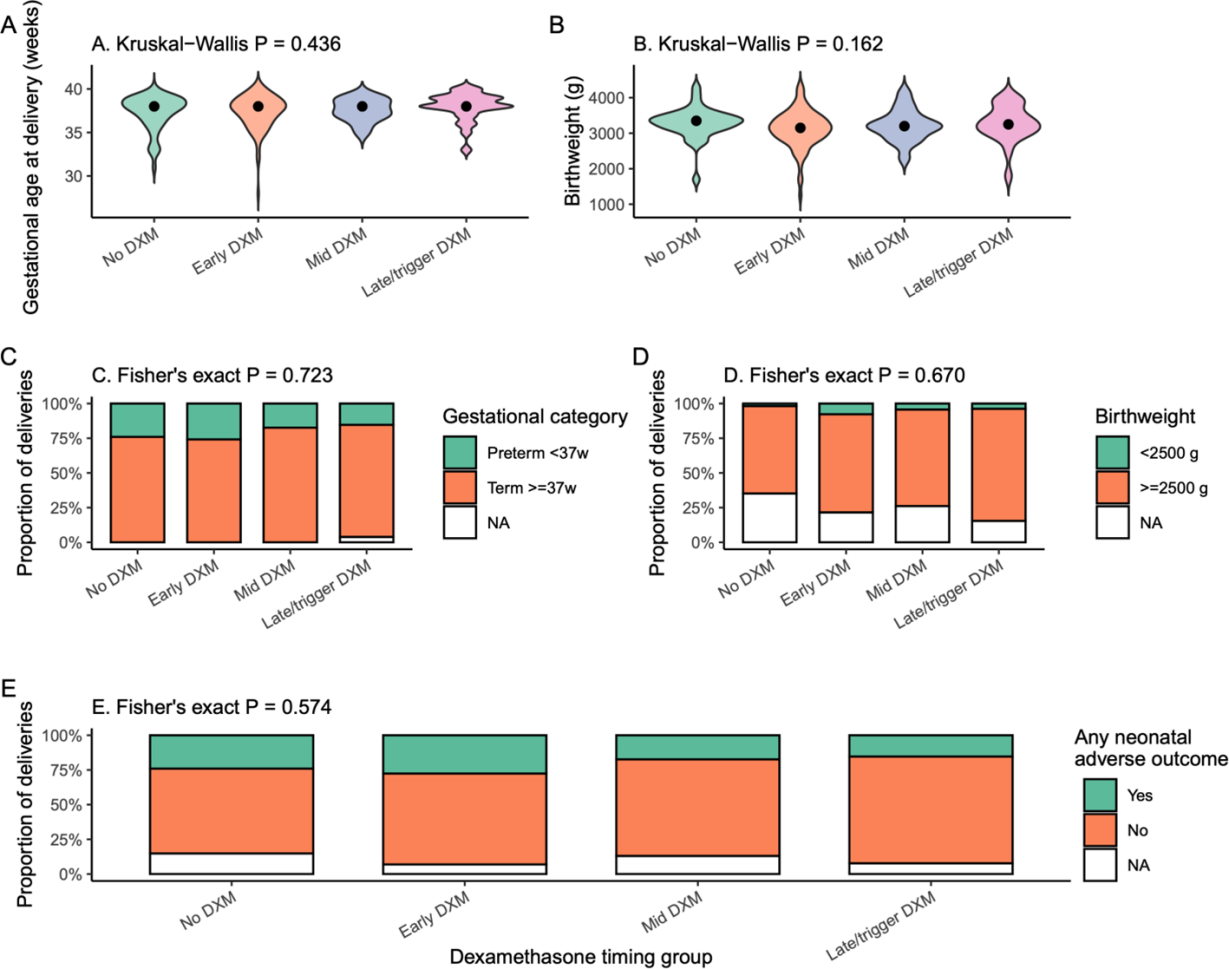
Neonatal and perinatal outcomes according to dexamethasone timing. **(A)** Gestational age at delivery (weeks) among cycles resulting in live birth, displayed as violin plots with median points and Kruskal–Wallis P value. **(B)** Birthweight (g) by DXM timing group, shown as violin plots with Kruskal–Wallis P value. **(C)** Proportion of deliveries that were preterm (<37 weeks) or term (≥37 weeks) in each group (stacked bars; Fisher’s exact P). **(D)** Proportion of deliveries with low birthweight (<2500 g) vs ≥2500 g (stacked bars; Fisher’s exact P). **(E)** Composite endpoint of any neonatal adverse outcome (preterm birth, low birthweight or congenital malformation) by DXM timing group (stacked bars; Fisher’s exact P). .

Neonatal outcomes among deliveries are summarized in **Table 5B** and **Figures 8A–E**. Gestational age at delivery was similar across all groups (mean ≈37–38 weeks; P≈0.48), with the vast majority of births occurring at term (≥37 weeks) (**Figures 8A–C**). Mean birthweight ranged from approximately 3.1 to 3.3 kg and did not differ significantly by DXM timing (P≈0.28; **Figure 8B**; **Table 5B**). The proportions of preterm birth (<37 weeks) and low birthweight (<2500 g) were low and comparable between groups (**Figures 8C, D**).

No congenital malformations were recorded in any group (**Table 5B**). When a composite endpoint of any neonatal adverse outcome (preterm birth, low birthweight or malformation) was examined, no significant differences were observed between DXM timing groups (**Figure 8E**). Taken together, these data suggest that varying the timing of DXM during controlled ovarian stimulation did not adversely affect neonatal outcomes in this cohort.

## Discussion

In this retrospective cohort study of 644 COS cycles, we systematically evaluated how the timing of dexamethasone (DXM) initiation during ovarian stimulation relates to ovarian response, embryo yield, live birth and neonatal safety, and several key findings emerged. First, compared with cycles without DXM, cycles exposed to DXM—particularly those with mid- and late/trigger–follicular initiation—demonstrated differences in stimulation characteristics and ovarian response, including shorter stimulation duration, lower overall Gn requirements, higher hCG-day E2 and progesterone concentrations, and greater oocyte and embryo yield in the full COS cohort. Second, across the ovarian response cascade, DXM timing appeared to exert its most consistent impact at the level of total usable embryos per cycle, with mid- and late DXM groups generating more transferable and frozen embryos and a higher probability of achieving ≥3 or ≥5 usable embryos than cycles without DXM. Third, at the clinical end of the spectrum, within the post-transfer cohort, early- and mid-follicular DXM were associated with higher live-birth rates, even after multivariable adjustment and in propensity score–weighted models, whereas late/trigger DXM did not confer a clear benefit. Fourth, mediation analysis did not provide statistically significant evidence that total usable embryos mediated the association between early DXM and live birth, although the direct effect remained statistically significant. Finally, we observed no evidence of harm with respect to gestational age at delivery, birthweight, preterm birth, low birthweight or congenital malformations, and a composite adverse neonatal endpoint was comparable across groups. Taken together, these findings suggest that DXM timing matters: mid- and late/trigger DXM were associated with higher usable embryo yield, whereas early- and mid-follicular DXM were associated with more favorable live-birth estimates in the post-transfer cohort. Late/trigger DXM did not show a clear live-birth advantage.

Glucocorticoids have long been used empirically in assisted reproduction, particularly in women with autoimmune disease, thyroid autoimmunity or suspected immune-mediated implantation failure. Prior studies evaluating prednisolone or DXM have produced heterogeneous and often conflicting results, with some suggesting improved implantation or clinical pregnancy in selected subgroups and others reporting no clear benefit over standard care. Many of these studies, however, have been limited by small sample sizes, heterogeneous inclusion criteria, variable dosing and timing regimens, and a predominant focus on intermediate endpoints such as biochemical or clinical pregnancy rather than live birth (10–18). Moreover, most prior work has treated glucocorticoid use as a simple binary exposure (yes/no), without explicitly considering when during COS glucocorticoids are introduced.

By contrast, our study explicitly decomposed DXM exposure into early-, mid- and late/trigger–follicular windows and examined a broad outcome spectrum from stimulation parameters through embryo metrics to live birth and neonatal outcomes. The observation that mid and late/trigger DXM were associated with a higher number of usable embryos, whereas early- and mid-follicular DXM were associated with higher odds of live birth, is biologically plausible (10–12, 14, 16, 17). Early follicular glucocorticoid exposure may modulate intra-ovarian cytokine networks, influence follicular recruitment and synchronization, or impact granulosa cell steroidogenesis in ways that ultimately favor oocyte competence. Mid-follicular exposure may exert more subtle effects on follicle maturation and ovulatory readiness. In contrast, initiation only in the late follicular phase or at trigger may be too late to substantially influence oogenesis or early embryogenesis, while still exposing the endometrium and early gestation to glucocorticoids without clear benefit.

Our mediation analysis provides additional nuance: although the direct association between early DXM and live birth remained statistically significant, the indirect effect through total usable embryos was not statistically significant. These findings suggest that the observed live-birth association was not strongly explained by embryo number alone and may involve other biologic or clinical pathways. Together with the ΔP sensitivity analyses (**Supplementary Table 8**), these data suggest that late-follicular progesterone dynamics may partly contribute to differences in embryo yield, but do not fully explain the observed associations between DXM timing and live birth.

From a practical standpoint, our findings do not support indiscriminate glucocorticoid use in all IVF cycles. At our center, DXM was used as a selective, clinician-directed adjuvant, mainly when current-cycle monitoring suggested a higher risk of premature progesterone elevation or endocrine/inflammatory dysregulation during COS. The treated population therefore should not be viewed as a broadly defined IVF population, but as a clinically selected group characterized by follicular development pattern, ovarian responsiveness to gonadotropins and dynamic serum progesterone changes. Within this context, initiating DXM earlier in the stimulation course may be more rational than deferring it to the peri-trigger window, because earlier exposure could plausibly influence follicular synchronization, the ovarian inflammatory milieu and steroidogenic regulation before late follicular events are established. Early and mid DXM were associated with more favorable live-birth estimates, whereas increased usable embryo yield was more evident for the mid and late/trigger groups. Thus, a ‘late-only’ strategy appears hard to justify based on our data. It is important to emphasize, however, that DXM should not be viewed as a universal ‘booster’ for all patients; careful selection and monitoring remain essential.

The subgroup analyses by age and AMH, although exploratory, are also informative. The suggestion that early DXM may be more beneficial in younger women and those with higher AMH is consistent with the idea that modulating the micro-environment may have greater impact when there is an underlying capacity for robust follicular and embryo output. In contrast, in women with diminished ovarian reserve or advanced reproductive age, glucocorticoids may be less able to overcome intrinsic limitations in oocyte number and quality. These findings argue against a one-size-fits-all strategy and support a more individualized, phenotype-based approach to DXM use.

Any potential benefit of glucocorticoid use in IVF must be weighed against safety concerns for both the mother and the offspring. Glucocorticoids cross the placenta and have been associated, in other contexts, with altered fetal growth and neurodevelopment when used at high doses or for prolonged periods. In our cohort, we did not observe significant differences in gestational age, birthweight or major neonatal morbidity across DXM timing groups; however, our study was not powered to detect rare outcomes or long-term effects (27–30, 35, 36). A composite endpoint of any neonatal adverse outcome was also similar between groups. These findings are reassuring and suggest that, within the low-dose oral regimen used in our practice (0.75-1.125 mg once daily, predominantly 0.75 mg/day), DXM did not adversely affect neonatal outcomes. However, our study was not powered to detect very rare events, and longer-term follow-up of offspring was not available; thus, subtle or late-emerging effects cannot be excluded. Prospective registries and linkage to pediatric outcomes would be valuable to further refine the safety profile of adjuvant glucocorticoids in IVF.

Our study has several methodological strengths. We leveraged a relatively large single-center cohort with detailed stimulation, laboratory and outcome data, allowing granular characterization of ovarian response, embryo metrics and live birth. Rather than treating glucocorticoid exposure as a simple yes/no variable, we defined clinically meaningful timing windows for DXM initiation and related these to a broad spectrum of endpoints. We adjusted for key confounders (age, BMI, AMH, AFC and embryos transferred) using multivariable regression and further addressed confounding by indication with propensity score–based inverse probability of treatment weighting (IPTW). Covariate balance after weighting was carefully checked using standardized mean differences, and effect estimates were generally consistent between conventional and IPTW-weighted models, which increases confidence that our findings are not solely artifacts of baseline imbalance. By additionally incorporating mediation analysis, we moved beyond simple association toward a more mechanistic understanding of how early DXM might influence live birth, highlighting total usable embryos as a clinically relevant mechanistic candidate, although formal mediation through this pathway was not statistically significant. Overall, the combination of regression, PS/IPTW, subgroup and mediation analyses provides a more robust and nuanced perspective than any single approach alone.

Nonetheless, several limitations warrant consideration. The retrospective, single-center design inherently limits causal inference, and although we used multivariable adjustment and PS/IPTW, residual confounding from unmeasured or imprecisely measured factors cannot be excluded. In particular, because DXM use was driven by the treating physician’s assessment of current-cycle follicular development, ovarian responsiveness, and evolving progesterone levels, the DXM groups were clinically selected rather than randomly assigned. This creates a meaningful risk of selection bias and confounding by indication: patients prescribed DXM may have differed from untreated patients in the perceived risk of premature progesterone elevation, follicular asynchrony, endocrine instability, or inflammatory status. We attempted to mitigate this risk through multivariable adjustment, diagnosis-adjusted sensitivity analyses, ΔP-adjusted analyses, and PS/IPTW, and measured baseline demographic, infertility and ovarian reserve characteristics were broadly balanced; however, unmeasured or imprecisely captured indications remain possible. Detailed histories of previous miscarriage, recurrent implantation failure, immune abnormalities, and structured DXM-indication fields were unavailable in the analytic dataset; these factors may have influenced both prescribing decisions and reproductive outcomes. Our classification of DXM timing into early, mid and late/trigger windows was based on initiation day only. However, dominant follicle diameter at DXM initiation differed markedly across the treated timing groups and was highly concordant with the expected follicular developmental stage, supporting the biological coherence and reproducibility of the day-based exposure definition (**Supplementary Table 9**). Although the institutional dose range was known (0.75-1.125 mg/day, predominantly 0.75 mg/day), individual-level dose, adherence, exact stop date and cumulative exposure were not captured in a structured manner suitable for formal dose-response analysis, making it difficult to fully separate the effects of timing from those of exposure intensity. We analyzed live birth in the post-transfer cohort rather than cumulative live birth across all fresh and subsequent frozen embryo transfers arising from a given stimulation cycle. Accordingly, our data do not provide a true cumulative started-cycle live-birth endpoint, and the full downstream impact of increased embryo yield may therefore be underestimated. The study was also underpowered to detect very rare obstetric or neonatal complications or to assess longer-term child outcomes. Finally, both the decision to use DXM and the dosing regimen reflect local protocols at a single tertiary center, and the generalizability of our findings to other settings may be limited.

Taken together, our findings support a more nuanced view of glucocorticoid use in IVF. Rather than asking only whether DXM is “beneficial,” our data suggest that when DXM is started during COS—and in which patients—matters. Mid- and late/trigger DXM were associated with increased embryo yield, whereas early- and mid-follicular DXM were associated with higher odds of live birth without detectable detriment to neonatal outcomes. Late/trigger DXM did not show a clear live-birth advantage. These results should not be interpreted as a blanket endorsement of DXM for all IVF patients, but rather as a rationale for more targeted use in individuals for whom glucocorticoids are already being considered. Prospective, ideally randomized, trials stratifying DXM timing and incorporating detailed immunologic and endocrine profiling will be needed to confirm these observational findings and further delineate the mechanisms by which DXM influences ovarian response and implantation. Integration of cumulative live birth, long-term offspring outcomes and cost-effectiveness analyses will help determine the overall value of DXM as an adjuvant in modern IVF programs.

## Conclusion

In conclusion, in this retrospectively analyzed cohort, mid- and late/trigger DXM use during COS was associated with a modest increase in total usable embryos, whereas early- and mid-follicular DXM were associated with a higher likelihood of live birth in the post-transfer cohort without obvious compromise of neonatal safety. Late/trigger DXM did not provide a clear live-birth advantage. These data provide a framework for more evidence-informed, timing-sensitive use of dexamethasone in IVF and highlight the importance of considering both embryo yield and live birth, rather than intermediate endpoints alone, when evaluating adjuvant therapies in assisted reproduction.

## Data Availability

The raw data supporting the conclusions of this article will be made available by the authors, without undue reservation.
