# Supplementary material for: Timing of dexamethasone initiation during controlled ovarian stimulation and live birth after IVF/ICSI: a retrospective cohort study
Source: Front Endocrinol (Lausanne). 2026 May 13;17:1764227. doi: 10.3389/fendo.2026.1764227 (PMC13181565; doi:10.3389/fendo.2026.1764227)
Supplement: Supplementary file 1 [file DataSheet1.pdf]

Supplementary Legends

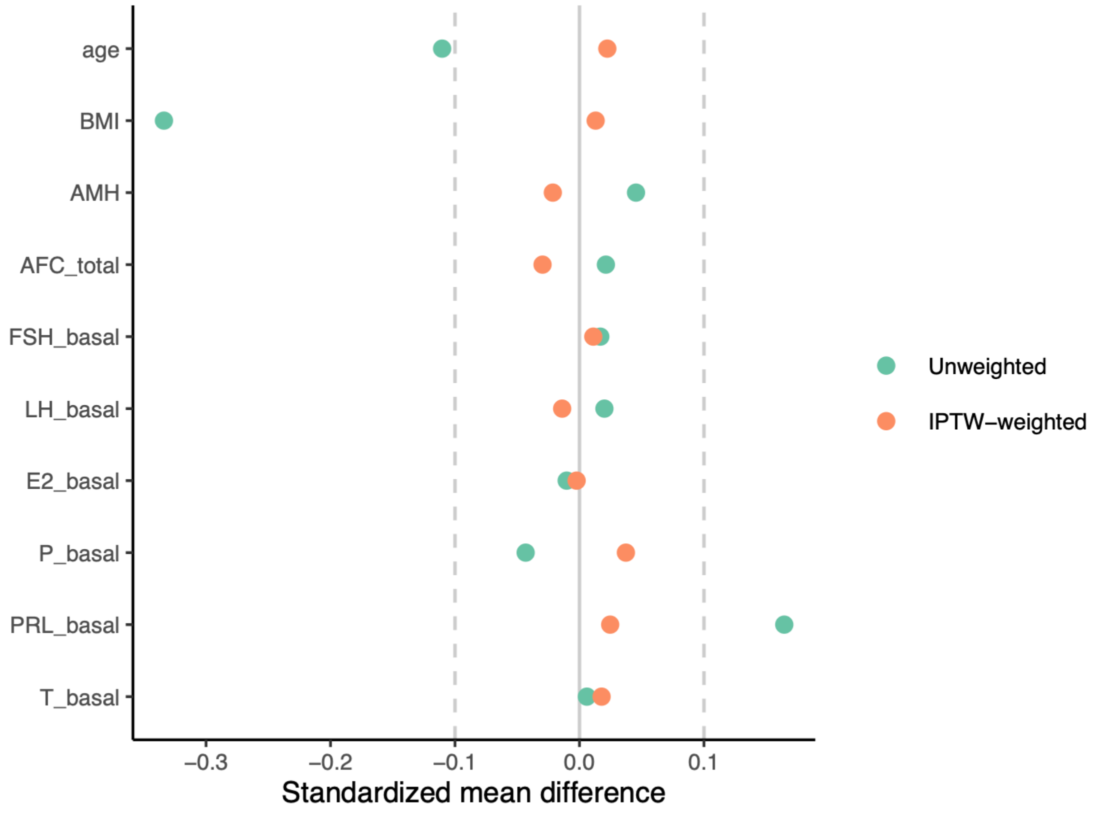

Supplementary Figure S1. Covariate balance for any dexamethasone use versus no dexamethasone.

Standardized mean differences (SMD) for baseline covariates before (unweighted) and after inverse probability of treatment weighting (IPTW) in the comparison of any dexamethasone (DXM) versus no DXM. Each point represents the SMD for a single covariate (age, BMI, AMH, AFC, and basal FSH, LH, E2, P, prolactin and total testosterone). Dashed vertical lines at SMD = ±0.10 indicate the threshold for acceptable covariate balance.

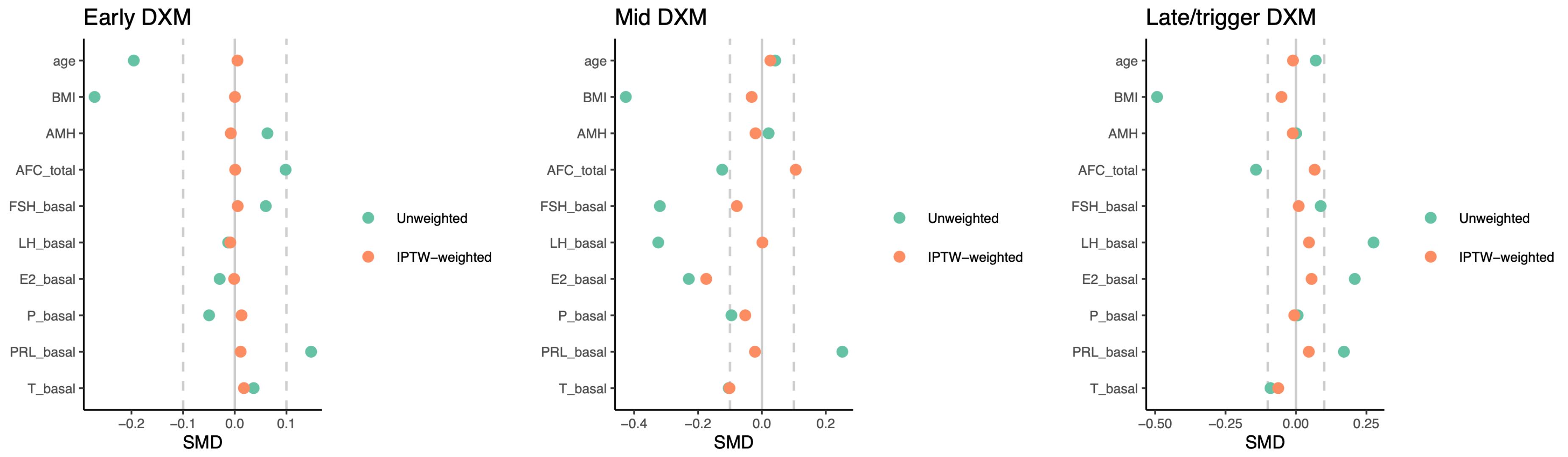

Supplementary Figure S2. Covariate balance for early, mid and late/trigger dexamethasone versus no dexamethasone.

Standardized mean differences (SMD) for baseline covariates before (unweighted) and after IPTW in pairwise propensity score models comparing (A) early DXM versus no DXM, (B) mid DXM versus no DXM and (C) late/trigger DXM versus no DXM. Points show SMDs for age, BMI, AMH, AFC and basal FSH, LH, E2, P, prolactin and total testosterone. Dashed vertical lines at SMD = ±0.10 denote the threshold for acceptable balance.

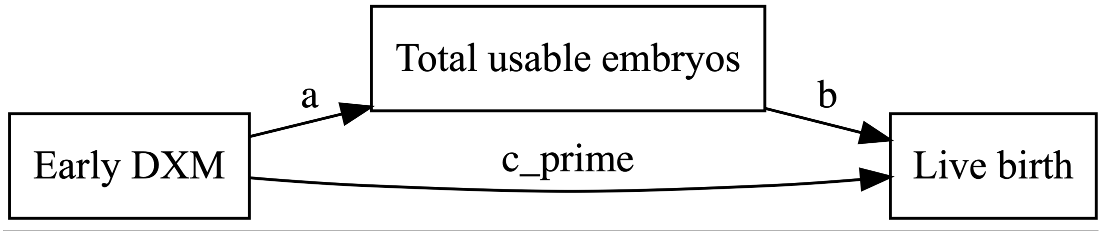

Supplementary Figure S3. Conceptual mediation model for early dexamethasone and live birth.

Directed acyclic graph illustrating the hypothesized mediation pathway whereby early-follicular DXM exposure influences live birth both directly and indirectly through total usable embryos. Paths are labelled a (effect of early DXM on total usable embryos), b (effect of total usable embryos on live birth) and c′ (direct effect of early DXM on live birth after accounting for the mediator).

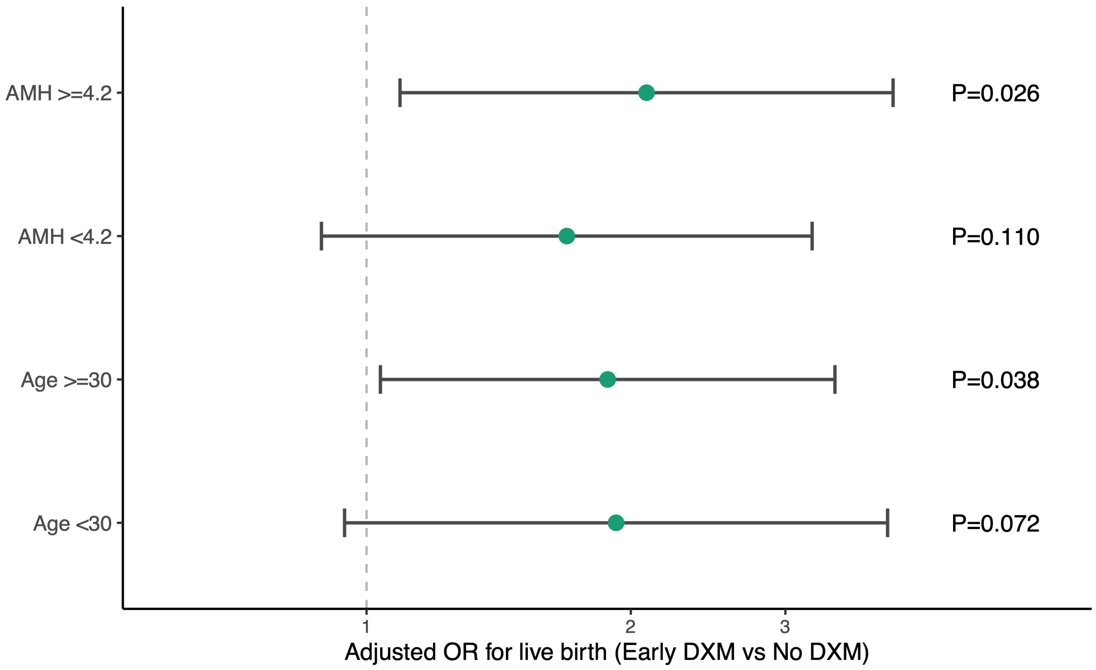

Supplementary Figure S4. Subgroup analysis of early dexamethasone versus no dexamethasone for live birth.

Forest plot of adjusted odds ratios (ORs) for live birth comparing early DXM versus no DXM within strata defined by age (<30 vs ≥30 years) and AMH (<4.2 vs ≥4.2 ng/mL). Models are adjusted for age, BMI, AMH, AFC and number of embryos transferred. Horizontal bars represent 95% confidence intervals; P values for each stratum are shown to the right of the plot.

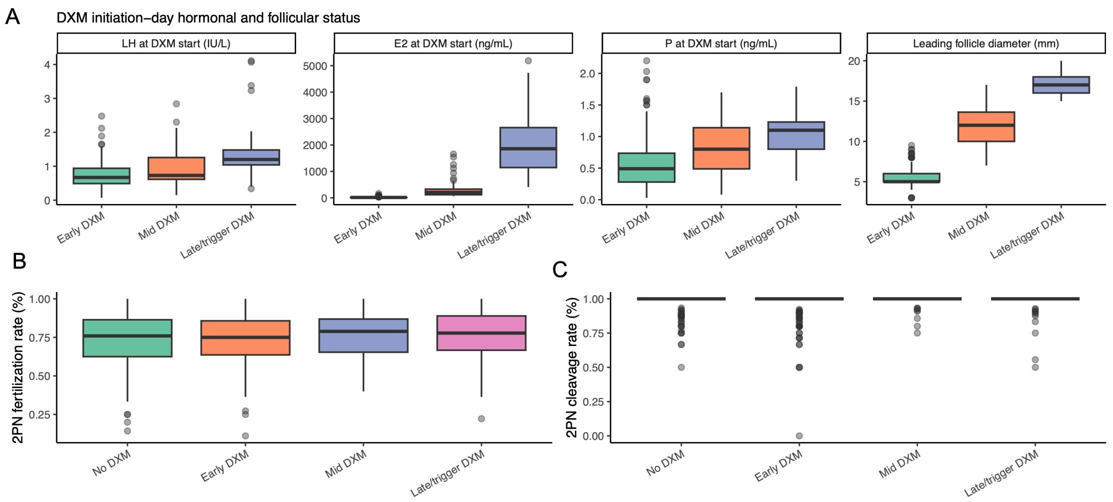

Supplementary Figure S5. Dexamethasone initiation-day hormonal and follicular status and 2PN quality metrics.

(A) Boxplots of LH, E2 and P concentrations at the time of DXM initiation and leading follicle diameter (mm) at initiation in cycles with early, mid and late/trigger DXM. (B) Boxplots of 2PN fertilization rate (2PN zygotes/total oocytes, %) across no DXM, early, mid and late/trigger DXM groups. (C) Boxplots of 2PN cleavage rate (cleaved 2PN embryos/2PN zygotes, %) across the same groups.

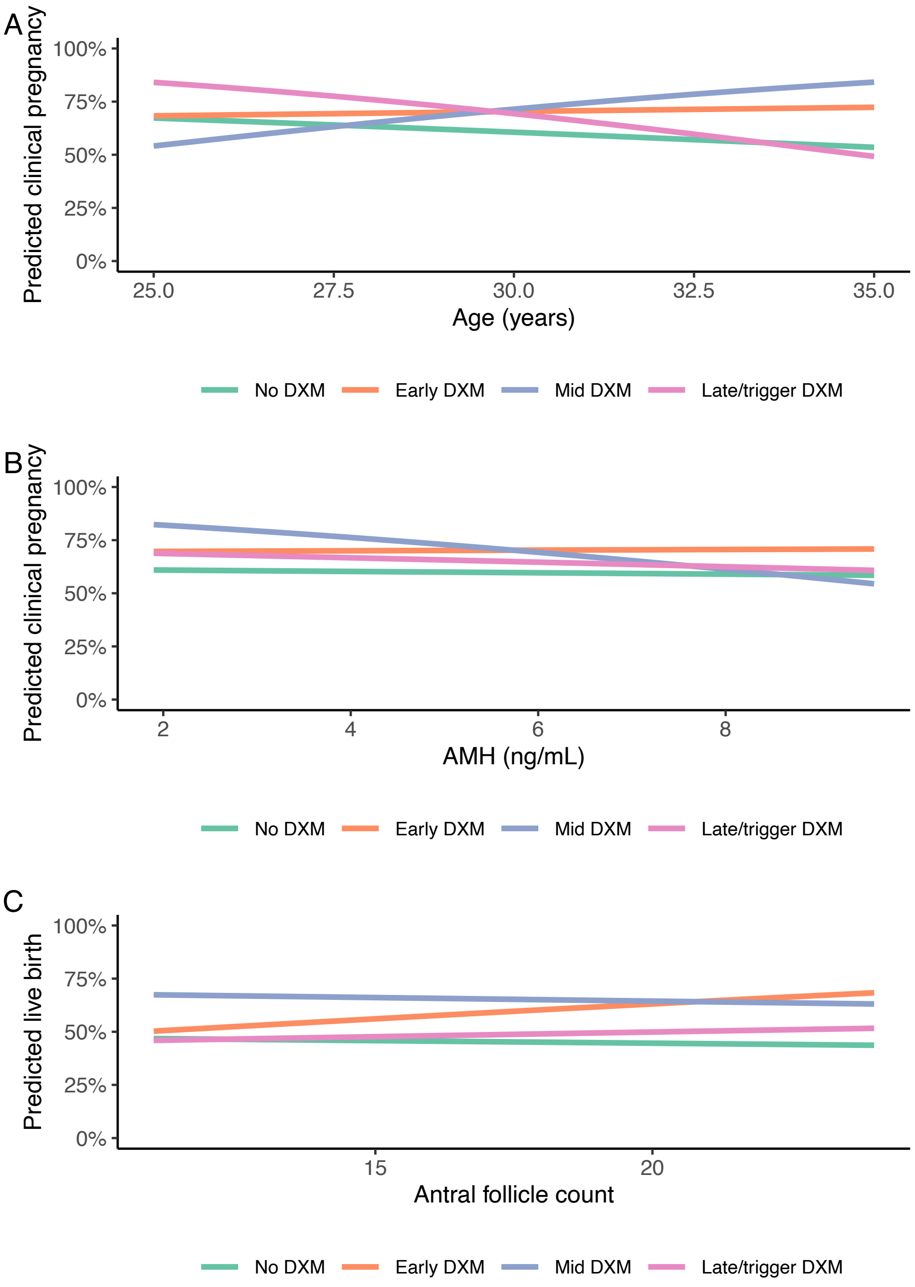

Supplementary Figure S6. Predicted probabilities of clinical pregnancy and live birth in the post-transfer cohort according to age, AMH and AFC.

Predicted probabilities from multivariable logistic regression models fitted in the post-transfer cohort showing (A) clinical pregnancy versus age, (B) clinical pregnancy versus AMH, and (C) live birth versus antral follicle count (AFC) for each DXM timing group. Predictions were generated while holding other covariates at their median values; curves are displayed as percentages on the y-axis.

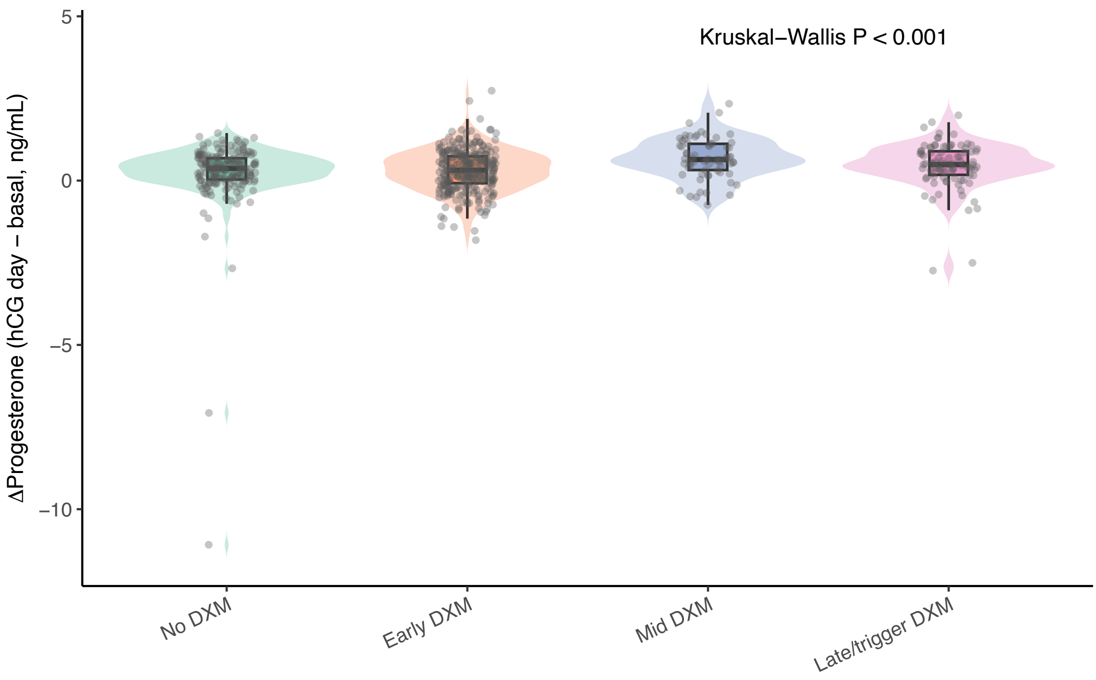

Supplementary Figure S7. Distribution of within-cycle progesterone change according to dexamethasone timing.

Violin plots with overlaid boxplots and individual cycles show within-cycle progesterone change (ΔP), defined as hCG-day progesterone minus basal progesterone, across the no DXM, early DXM, mid DXM, and late/trigger DXM groups. The overall P value was obtained using the Kruskal–Wallis test.
